# Psychometric Properties and Reliability of the Referee Self-Efficacy Scale (REFS) in Volleyball Referees

**DOI:** 10.3390/ijerph17228423

**Published:** 2020-11-13

**Authors:** Pierluigi Diotaiuti, Lavinia Falese, Stefania Mancone, Stefano Corrado, Luca Mallia, Arnaldo Zelli, Fabio Lucidi

**Affiliations:** 1 Laboratory of Epidemiology, Physical Activity and Lifestyles, Department of Human Sciences, Society and Health, University of Cassino and Southern Lazio, 03043 Cassino, Italy; l.falese@unicas.it (L.F.); s.mancone@unicas.it (S.M.); stefano.corrado@unicas.it (S.C.); 2 Department of Movement, Human and Health Sciences, University of Rome “Foro Italico”, 00135 Rome, Italy; luca.mallia@uniroma4.it (L.M.); arnaldo.zelli@uniroma4.it (A.Z.); 3 Department of Developmental and Social Psychology, Sapienza University of Rome, 00185 Rome, Italy; fabio.lucidi@uniroma1.it

**Keywords:** volleyball, referees, self-efficacy, confirmatory factor analysis

## Abstract

Background: Volleyball officials require a combination of qualities, e.g., confidence, decisiveness, courage and mental toughness as very important attributes for their performance. Measurement of the self-efficacy of volleyball referees has not been studied with large samples; therefore, the aim of this study was to fill this gap in the research. Methods: Four-hundred and forty-five international volleyball referees participated in the study fulfilling the referee self-efficacy scale in the English version. Results: The confirmatory analysis verified the four-factor structure of the scale and its reliability in this specific sample of international volleyball referees. ANCOVA revealed a significant effect of the covariate “level of education” for all four dimensions of referees’ self-efficacy. Conclusions: Although English was not the mother tongue for most of the subjects in the sample, the scale was statistically reliable, and the items were easy to understand, thus making the tool very suitable to use for further studies on multilingual referees. The study also suggests enhancing the level of education of the officials because of its significant effect on the perceived self-efficacy during refereeing.

## 1. Introduction

Sports officials are important key actors in any sport competition; however, they have often under-researched by the scientific literature [1]. Referees are subjected to physical and psychological stressors due to the many aspects of a game/match they are officiating, such as decision-making processes, communication with athletes and staff, managing the game, nature of the spectators and pressure in general [1,2,3].

In addition to aspects related to physical preparation, experience and psychological factors are also very important for a good performance by the sports referee [4]. The mistakes made in the officiating tasks and the pressure from the players, staff and spectators can lead to high levels of stress, anxiety, low self-confidence and often lead to referees drop out [1,5,6].

Years of experience have been found positively associated with decision-making skills in football referees [2,7]; however, some studies have found that the influence of the experience on the referee’s performance may depend on the type of sport [4,8].

Previous studies identified self-efficacy, defined by Bandura as a judgment about one’s capability to successfully perform a task, as an important factor influencing the performance, behaviors, satisfaction and stress in sports officials [1,9]. According to Myers et al. [10], referee self-efficacy is composed of four dimensions that can be assessed through a 13 items scale (referee self-efficacy scale—REFS): game knowledge, decision-making, pressure and communication [10].

The knowledge of the game is represented by the confidence that the referees have in relation to their knowledge of the rules of the sport and its strategies [10]. The knowledge of the rules comes from study, preparation and experience, but it is essential that the referee is also able to apply the rules during a match [11].

The decision-making process within REFS corresponds to the referee’s confidence in making decisions quickly and firmly. The referees who manage the mental process correctly will be more successful in decision-making and thinking styles [12].

Pressure from players, staff and spectators is also an element influencing the self-efficacy of a referee and, consequently, his behaviors and performance. Referees who are exposed to pressure from spectators, players, staff during and after a competition and who do not have a strong character and good self-esteem have difficulty performing and risk making a greater number of mistakes [13].

The last dimension identified in the REFS is communication with players, staff and other referees. When the referee has good communication skills with the other actors of the competition, his/her self-efficacy increases and indirectly also his/her performance [14].

The REFS tool, as far as we know, has been used previously in studies about team sports such as football, basketball, handball [13,15,16]. In the abovementioned studies, REFS total scores and, more specifically, levels of perceived game knowledge and decision-making skills show significant differences according to gender, level of education and level of experience, but these findings are not always confirmed in other studies [17]. The samples used in the mentioned studies included below 200 participants, and all the participants were speaking the same language, contrary to our study on volleyball referees.

Volleyball is a team sport generally officiated by two referees standing on opposite sides. The first referee stands on a highchair and is know as head referee or up referee, while the second referee stands at a lower level and has the role of assistant. The pair of referees, contrary to what happens in other sports such as handball, can change in every match, and the referees can be either first or second referee according to the decision of the National or International Federation.

The referees need to stand for long periods of time, but the game is the result of fast action in a small area (18 × 9 mt). For this reason, they must maintain high levels of focus and alertness without missing any detail and make calls positively and with good timing. Volleyball officials require a combination of qualities; some are innate, such as good instincts, fairness, mental toughness, confidence and decisive nature, while others can be developed with experiences such as attention to details, alertness and quick, but sound reactions [11].

Self-efficacy in volleyball referees has been studied in a sample of 76 US volleyball referees in 2015 in relation to performance; however, due to some limitations of the study, the results did not show a high significance of the relationship [4].

Therefore, the aim of this study was to fill a gap in the literature by verifying the four-factors structure of the referee self-efficacy scale (REFS), the relationships of the REFS dimensions with some demographic variables (gender, educational status, level of experience), as well as its reliability in a specific sample of referees, namely international volleyball referees.

## 2. Materials and Methods

### 2.1. Participants and Procedures

Four-hundred and forty-five international volleyball referees (82.2% male) aged between 25 and 66 years (mean age = 41.78; SD = 7.15) agreed to participate in the study and filled out an online questionnaire. Since a total of 1286 international referees of the International Volleyball Federation (Fédération Internationale de Volleyball—FIVB) were licensed at the time the questionnaire was administered through a contact mail, the sample of participants in our study was equivalent to 35% of the entire reference population.

Overall, they had experience as referees ranging from 3 to 43 years (mean years = 20.40; SD = 7.17). Table 1 reports the sociodemographic characteristics of the sample as well as their geographic area.

### 2.2. Measures

The 13-item version of the referee self-efficacy scale [10] (Myers et al. 2012) was used to evaluate self-efficacy in the participating international volleyball referees. The stem for all items is “*In the context of performing your referee job, how confident are you in your ability to* ...”.

Each item relates to one of the four dimensions of referee self-efficacy:

(1) game knowledge (3 items, e.g., “*Understand the basic strategy of the game*”);
(2) decision making (3 items, e.g., “*make critical decisions during competition*”);
(3) pressure (3 items, e.g., “*uninfluenced by pressure from players*”);
(4) communication (4 items, e.g., “*communicate effectively with coaches*”).

Participants were asked to rate their confidence with each challenge using a 5-point response scale ranging from 1 (“low”) to 5 (“high”). The REFS scale used for the research was in English, the official language used during international competition.

### 2.3. Data Analysis

**Figure 1:**
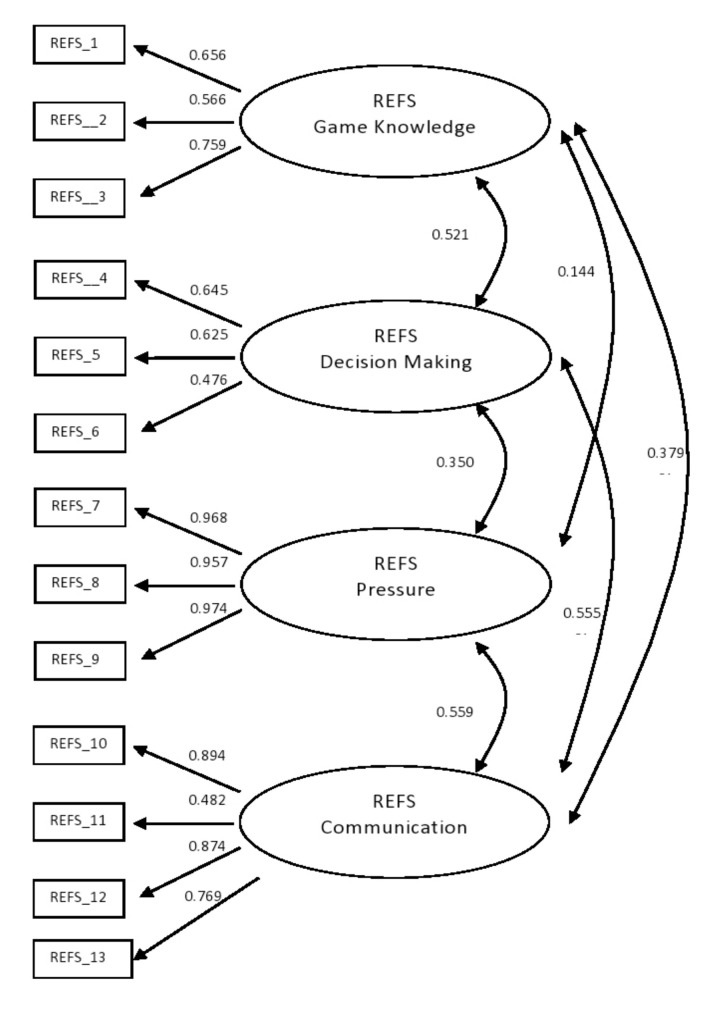
CFA Results.

Confirmatory factor analyses (CFA) of the referee self-efficacy scale were conducted using MPLUS software (Version 7) [18]. The CFA was conducted using data from the entire sample (*n* = 445). This CFA examined the measurement hypothesis that each item loaded only on one of the four factors described by the original validating study. This measurement model is depicted in Figure 1. Model parameters were estimated using the maximum likelihood (ML) estimation method, and the quality of the measurement model was visually examined through the fit indices estimates of CFI (comparative fit index), RMSEA (root mean square error of approximation) and SRMR (standardized root mean square residual) [19]. Cutoff values of 0.90 or above for the CFI indicated acceptable models, although values greater than 0.95 were preferable [19]. Values of 0.08 or less for the RMSEA and the SRMR were deemed satisfactory for well-fitting models [19]. Cronbach’s alpha coefficients for each subscale were estimated to evaluate their reliability.

Four ANCOVAs considering the mean scores on the four scales as dependent variables were carried out in order to ascertain gender differences and, controlling for the effects of the education level, the years of experience as a referee were considered as covariates. Finally, bivariate correlations between the four subscales and education level, as well as with the years of experience as a referee, were also estimated.

## 3. Results

### Factorial Structure and Reliability of the Referee Self-Efficacy Scale

The CFA conducted on the entire sample of volley referees showed that the four-factor model of the scale fit the data very well (χ^2^_(59)_ = 137.527, *p* < 0.001; CFI = 0.98; RMSEA = 0.055; 90% CI: from 0.043 to 0.067; SRMR = 0.044), that the four sets of items loaded significantly on their corresponding latent factor, and that the four latent factors were significantly correlated with each other. Figure 1 shows the details of these results.

Instead, Table 2 shows the reliability of each subscale of the REFS, as well as their descriptive statistics. In agreement with George and Mallery’s findings [20], neither of the four scales we analyzed showed unacceptable alphas values (i.e., ≤0.5). More specifically, one scale (i.e., pressure) showed an “excellent” value (i.e., ≥0.90), one scale (i.e., communication) a “good” value (i.e., ≥0.80), and the other two scales (i.e., decision making and game knowledge, respectively) showed values ranging from “acceptable” (i.e., ≥0.70) to “poor” (i.e., ≥0.50).

Overall, the responses in all four subscales showed a slight negative skewness, probably due to the selected sample (i.e., all international level referees).

As reported in Table 3, the ANCOVAs showed a significant difference across gender only for “game knowledge”, since the male referees are more confident about this issue than female referees. The same pattern also emerged for the “decision-making” dimension even though the difference only approaches statistical significance. Furthermore, the covariate “level of education” resulted significantly related to all four dimensions. In particular, as reported in Table 4, a higher level of education in volleyball referees is significantly associated with higher levels in all four dimensions of referees’ self-efficacy. However, in particular were more evident, considering both levels of significance and effect sizes, the associations with the perception of communicative efficacy (*p* < 0.001; *Eta^2^* = 0.049) and the tolerance of the competitive pressure (*p* = 0.001; *Eta^2^* = 0.026). Finally, with respect to the covariate “years of experience”, the ANCOVA revealed a significant effect only for “game knowledge” and “communication” dimensions. As reported in Table 4, greater experience was related to a higher perception of self-efficacy in these two dimensions.

## 4. Discussion

The aim of this study was to verify the psychometric properties and the reliability of the four-factors structure of the referee self-efficacy scale (REFS) in international volleyball referees. The present study showed that the referee self-efficacy scale fits well with the data of the international volleyball referees and that the four dimensions of the scale were significantly interrelated.

Although English was not the mother tongue for most of the subjects in the sample, the scale resulted statistically reliable, and the items were very easy to understand, thus making this tool very useful and easy to use for further studies on multilanguage referees. Testing the self-efficacy of sports referees is very important in order to understand the influence of the different dimensions in the officiating performance outcomes and to suggest specific interventions.

Apart from the original study that tested the validity of the REFS scale on multisport referees in the US and Spain [10], the psychometric properties of the REFS have not been further assessed. A Spanish version of the scale has been tested on multisport referees, but, to our knowledge, this scale has never been tested on international volleyball referees [21].

With regard to the analysis of the relationship between gender and REFS dimensions, we found differences between male and female referees in relation to “game knowledge” and “decision-making”. The data showed that male referees recorded more conviction about the knowledge of the rules, the understanding of the basic strategy and the mechanisms of the game. Our results are in part similar to the one presented in a study on the self-efficacy of basketball referees showing that male referees gave a higher rating of their ability to make decisions in critical situations compared to female referees [13].

The results of the analysis also showed that a higher level of education in international volleyball referees was significantly associated with higher levels on all four dimensions of referees’ self-efficacy. This result does not agree with the findings of Karaçam et al. [13], who did not find any association between education and self-efficacy in basketball referees; however, it is in line with the results of a study on football referees determining that the level of self-efficacy and game knowledge of the football referees with a bachelor’s degree was higher than that of football referees with high school degree [15].

With regard to years of experience, instead, the bivariate correlations showed a positive association with the referees’ self-efficacy, especially in the dimension of game knowledge. The volleyball referees with more years of experience in officiating feel more confident in understanding the rules and the strategies of the games, and consequently, their general self-efficacy resulted in higher.

According to Dereceli et al. [15], the years of experience increase decision-making ability, but this is not evident in our analysis. Our results confirm the findings of previous studies that showed a positive association between the referee experience and the level of referees’ self-efficacy [14,15,22], but not the one in the research of Dereceli on the self-efficacy of football referees [15] and on the study on US volleyball referees [4].

A slight limitation of the study can be found in relation to the sample. Even though the total sample represents 35% of the total licensed international referees’ population, in the demographical characteristics table, it can be noticed that data from the geographical area of Australia are few (5), but from a deeper analysis of the population, we found that the total sample of the Australian international volleyball referees at that time was 16 so the significance of the sample is not compromised. Future research should analyze more specifically the relationship between self-efficacy, tested with the REFS, decision-making speed and the quality of the volleyball referees’ performance, while an undeniable merit of the study was that it succeeded in involving 35% of the entire international population of volleyball referees in the reliability assessment of the REFS.

## 5. Conclusions

The results of this study could suggest the use of REFS for sport scientists and sport managers in order to develop programs for volley referees, also of different nationalities and mother tongues and in order to facilitate the assessment and recruitment process. The findings revealed that there is an association between self-efficacy dimensions and some demographic variables such as gender, level of education and level of experience. For this reason, it is recommended that the managers responsible for refereeing matters for sports organizations and events take into consideration the different variables in order to maximize the implemented process of referee selection, training and assignments. The confirmatory analysis verified the four-factor structure of the scale and its reliability in this specific sample of international volleyball referees. Although English was not the mother tongue for most of the referees in the sample, the scale was statistically reliable, and the items were easy to understand, thus making the tool very suitable to use for further studies on multilingual referees.

## Figures and Tables

**Table 1:** Sociodemographic characteristics of the sample.

Variables		*N*	(%)
Gender	Males	366	82.2
	Females	79	17.8
Age	25–36 year	112	25.2
	37–41 year	120	26.9
	42–47 year	113	25.4
	48–66 year	100	22.5
Education	Middle	26	5.8
	High school graduation	115	25.8
	College degree	304	68.3
Level	International	429	96.4
	National	16	3.6
Experience	3–15 year	128	28.8
	16–20 year	128	28.7
	21–25 year	84	18.9
	26–43 year	105	23.6
Geographical Area	Mediterranean Europe	81	18.2
	Central Europe	96	21.6
	Baltic/Nord Europe	29	6.5
	Balkan Europe	94	21.1
	East Europe	34	7.6
	Middle East	30	6.7
	Asia	23	5.2
	Africa	33	7.4
	South America	20	4.5
	Australia	5	1.1

**Table 2:** Descriptive statistics and reliability of the four subscales of the referee self-efficacy scale (REFS).

	No. Items	Response Range	Mean (SD)	Skewness	Kurtosis	Cronbach’s Alpha
**Game knowledge**	3	1–5	4.62 (0.44)	−1.18	1.16	0.694
**Decision-making**	3	1–5	4.39 (0.58)	−1.01	1.11	0.593
**Pressure**	3	1–5	3.93 (1.42)	−1.23	−0.06	0.976
**Communication**	4	1–5	4.21 (0.78)	−1.27	1.53	0.840

**Table 3:** Mean score in REFS subscales across male and female referees.

			Gender		Level of Education		Years of Experience	
	Male (*n* = 366) Mean (SD)	Female (*n* = 79) Mean (SD)	F (*p*-Level)	Partial *Eta^2^*	F (*p*-Level)	Partial *Eta^2^*	F (*p*-Level)	Partial *Eta^2^*
**Game knowledge**	4.64 (0.43)	4.51 (0.47)	5.03 (0.022)	0.012	6.40 (0.012)	0.014	9.34 (0.002)	0.021
**Decision making**	4.41 (0.57)	4.28 (0.60)	3.58 (0.059)	0.008	7.63 (0.006)	0.017	0.62 (0.433)	0.001
**Pressure**	3.91 (1.45)	4.04 (1.31)	0.47 (0.493)	0.001	11.88 (0.001)	0.026	0.41 (0.524)	0.001
**Communication**	4.22 (0.79)	4.17 (0.81)	0.17 (0.685)	0.000	22.66 (<0.001)	0.049	4.69 (0.031)	0.011

**Table 4:** Bivariate correlations between REFS subscales, referees’ level of education and experience.

	Level of Education	Years of Experience
**Game knowledge**	0.096 *	0.149 *
**Decision making**	0.123 *	0.03
**Pressure**	0.162 *	0.01
**Communication**	0.210 *	0.08

* *p*-level < 0.05.

## References

[1] Guillén F., Feltz D.L. (2011). A conceptual model of referee efficacy. Front. Psychol..

[2] MacMahon C., Helsen W.F., Starkes J.L., Weston M. (2007). Decision-making skills and deliberate practice in elite association football referees. J. Sports Sci..

[3] Pietraszewski P., Roczniok R., Maszczyk A., Grycmann P., Roleder T., Stanula A., Ponczek M. (2014). The elements of executive attention in top soccer referees and assistant referees. J. Hum. Kinet..

[4] Spencer B.D. (2015). Self-Efficacy and Performance in Volleyball Referees. Master’s Thesis.

[5] Kaissidis-Rodafinos A., Anshel M.H., Porter A. (1997). Personal and situational factors that predict coping strategies for acute stress among basketball referees. J. Sports Sci..

[6] Rainey D., Winterich D. (1995). Magnitude of stress reported by basketball referees. Percept. Mot. Skills.

[7] Catteeuw P., Helsen W., Gilis B., Wagemans J. (2009). Decision-making skills, role specificity, and deliberate practice in association football refereeing. J. Sports Sci..

[8] Pizzera A. (2012). Gymnastic judges benefit from their own motor experience as gymnasts. Res. Q. Exerc. Sport.

[9] Bandura A. (1977). Self-efficacy: Toward a unifying theory of behavioral change. Psychol. Rev..

[10] Myers N.D., Feltz D.L., Guillén F., Dithurbide L. (2012). Development of, and initial validity evidence for, the referee self-efficacy scale: A multistudy report. J. Sport Exerc. Psychol..

[11] American Sport Education Program (2007). Officiating Volleyball.

[12] Arslanoğlu C., Doğan E., Acar K. (2018). Investigation of Decision Making and Thinking Styles of Volleyball Referees in Terms of Some Variables. J. Educ. Train. Stud..

[13] Karaçam A., Pulur A. (2017). Examining the relationship between referee self-efficacy and general self-efficacy levels of basketball referees in terms of certain variables. J. Educ. Train. Stud..

[14] Diotaiuti P., Falese L., Mancone S., Purromuto F. (2017). A structural Model of Self-efficacy in Handball Referees. Front. Psychol..

[15] Dereceli Ç., Ünlü H., Erbaş M.K. (2019). Investigation of Self-Efficacy Levels of Football Referees. Sak. Univ. J. Educ..

[16] Johansen B.T., Ommundsen Y., Haugen T. (2018). Referee efficacy in the context of Norwegian soccer referees—A meaningful construct?. Psychol. Sport Exerc..

[17] Eskiyecek C.G., Satici O., Ozaltas H.N., Savucu Y., Gul M. (2019). An Analysis on General Self-Efficacy Beliefs of Swimming Referees in Terms of Demographic Variables. J. Educ. Learn..

[18] Muthén L.K., Muthén B.O. (2012). Mplus User’s Guide.

[19] Hu L.T., Bentler P.M. (1999). Cutoff criteria for fit indexes in covariance structure analysis: Conventional criteria versus new alternatives. Struct. Equ. Modeling A Multidiscip. J..

[20] George D., Mallery P. (2003). Using SPSS for Windows Step by Step: A Simple Guide and Reference.

[21] Guillén F., Feltz D., Gilson T., Dithurbide L. (2019). Psychometric properties of the Spanish version of the Referee Self Efficacy Scale (REFS). Psicol. Deporte.

[22] Nazarudin M.N., Noordin H., Suppiah P.K., Abdullah M.R., Fauzee M.S.O., Abdullah N.M. (2014). Psychological skills assessment and referee rugby sevens performance. J. Pemikir Pendidik..

